# Supplementary material for: Clinical Course from Diagnosis to Death in Patients with Well-Differentiated Thyroid Cancer
Source: Cancers (Basel). 2020 Aug 18;12(8):2323. doi: 10.3390/cancers12082323 (PMC7463440; doi:10.3390/cancers12082323)
Supplement: Supplementary file 1 [file cancers-12-02323-s001.pdf]

cancers MDPI

Clinical Course from Diagnosis to Death in Patients with Well-Differentiated Thyroid Cancer

Hyunju Park, Jun Park, So Young Park, Tae Hyuk Kim, Sun Wook Kim, and Jae Hoon Chung

Table S1. Clinicopathological characteristics of DTC patients based on time interval.

Characteristics		1996-2002	2003-2009	2010-2018	*P*-value
**Number of Patients (Inoperable Patients)**		27 (2)	36 (3)	16 (1)	
Mean survival (month, SD)		74.37 (9.39)	56.25 (5.23).	63.19 (13.5)	
Sex (*n*, %)	Female	19 (70.4)	26 (72.2)	7 (43.8)	0.113
Primary tumor size (*n*, %)	<4cm	19 (70.4)	28 (77.8)	12 (75.0)	0.799
	4cm or more	8 (29.6)	8 (22.2)	4 (25.0)	
pT (*n*, %)	1	4 (16.0)	4 (12.1)	1 (6.7)	0.419
	2	5 (20.0)	3 (9.1)	1 (6.7)	
	3	11 (44.0)	11 (33.3)	8 (53.3)	
	4	5 (20.0)	15 (45.5)	5 (33.3)	
pN (*n*, %)	Not assessed	1 (4.0)	1 (3.0)	1 (6.7)	0.251
	N0	9 (36.0)	17 (51.5)	6 (40.0)	
	N1a	8 (32.0)	3 (9.1)	1 (6.7)	
	N1b	7 (28.0)	12 (36.4)	7 (46.7)	

SD, standard deviation; *n*, number of patients.

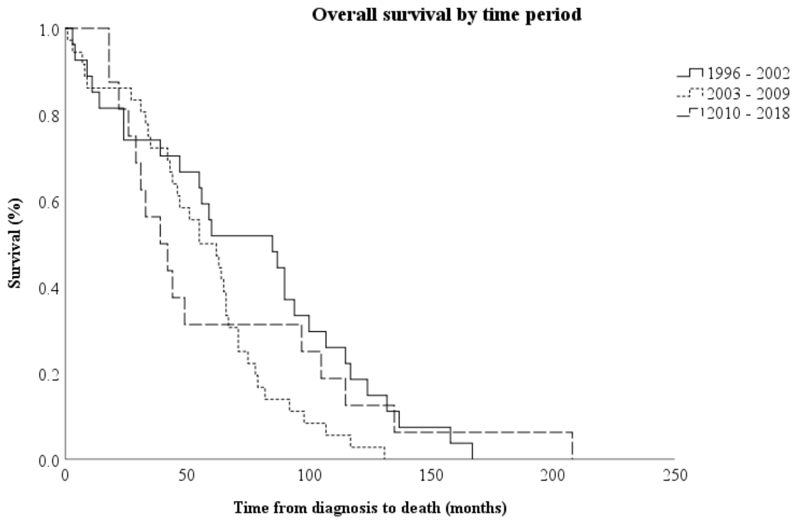

Figure S1. Overall survival based on time period.

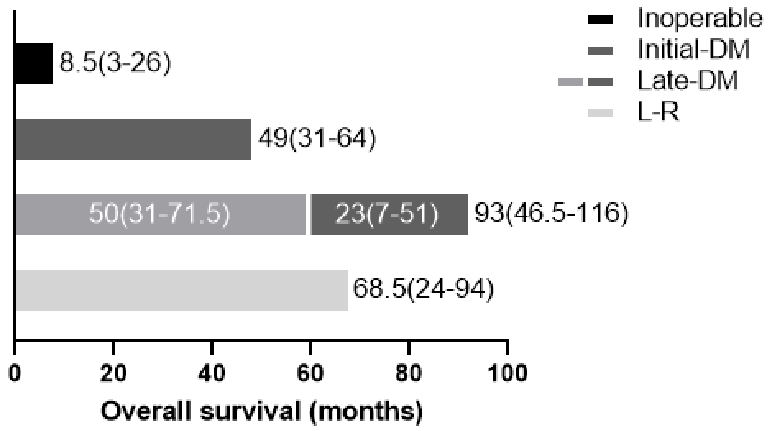

Figure S2. Timelines from diagnosis to death in each group. In late-DM, first column represented time from diagnosis to distant metastasis, and second column represented time from distant metastasis to death (median, interquartile range).

© 2020 by the authors. Licensee MDPI, Basel, Switzerland. This article is an open access article distributed under the terms and conditions of the Creative Commons Attribution (CC BY) license (http://creativecommons.org/licenses/by/4.0/).
